# Latent tuberculosis-related scleritis: a case report

**DOI:** 10.1186/s13104-016-2251-8

**Published:** 2016-09-20

**Authors:** Houssaine Ait Lhaj, Amine Benjelloun, Youssef bouia, Youssef Bennouk, Yassine Mouzari, Youssef El Kamouni, Mohamed Kriet

**Affiliations:** 1 Ophtalmology Unit, Avicenne Military Hospital, Marrakech, Morocco; 2 Pulmonology Unit, Avicenne Military Hospital, Marrakech, Morocco; 3 Biology Unit, Avicenne Military Hospital, Marrakech, Morocco

**Keywords:** Scleritis, Latent tuberculosis, Antitubercular therapy

## Abstract

**Background:**

Scleritis is a painful inflammatory process centered in the sclera that may involve the cornea and the underlying uvea. The etiology is commonly idiopathic or autoimmune but some cases are associated with systemic infection such as tuberculosis.

**Case presentation:**

In this report, we describe an unusual case of a female Moroccan patient who had a long history of bilateral recurrent scleritis associated with peripheral keratopathy and anterior uveitis. The patient was diagnosed with latent tuberculosis and responded to antitubercular therapy administrated after exclusion of other aetiologies. This patient was finally diagnosed with latent tuberculosis- related scleritis.

**Conclusions:**

Although systemic tuberculosis is reported as a possible cause of scleritis and other ocular inflammatory manifestations, assessment of the diagnosis of tuberculosis-related ocular inflammation is challenging especially in latent forms. The treatment is largely presumptive. However, a favorable response to antitubercular therapy without relapse is taken as evidence of the disease.

## Background

Scleritis is typically a severe painful inflammatory process centered in the sclera that may involve the adjacent tissues including the cornea and the underlying uvea [1]. It is a serious ocular condition that can lead to vision loss and therefore requires early diagnosis and treatment [2]. Scleritis is usually suspected from the clinical history, and confirmed by its characteristic clinical signs [1]. Fifty percent of patients with scleritis are diagnosed with an associated systemic disease including autoimmune conditions and infections [2]. Tuberculosis-related scleritis is an uncommon ocular inflammatory disorder that presents challenges in diagnosis and management for both ophthalmologists and infectiologists [3]. This report describes a case of bilateral recurrent scleritis associated with peripheral keratopathy and anterior uveitis as ocular manifestations of latent tuberculosis.

## Case presentation

A 43-year-old Moroccan female patient with a medical history of type 2 diabetes mellitus, presented with complaints of pain, redness and blurring of vision of her left eye since 3 days. She had a previous history of multiple episodes of scleritis occurring in both eyes over the last 5 years. During this time, the etiology of ocular inflammatory disorder remained unclear. Therapy consisted of oral non-steroidal anti-inflammatory drugs (NSAIDs) and topical steroids prescribed at every episode. Past ocular history also included a cataract surgery undergone on the left eye 2 years before. The patient, vaccinated at birth with BCG, reported a previous history of exposure to an active case of tuberculosis but denied any occurring feverish syndrome, night sweats, weight loss, shortness of breath or any other signs of systemic illness.

**Fig. 1:**
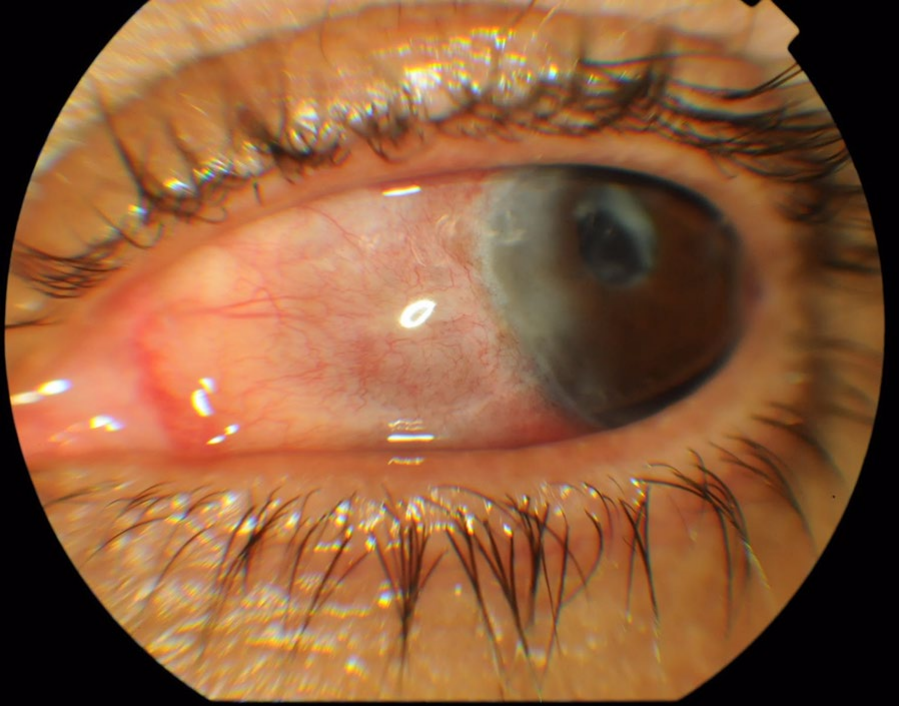
Cicatrised peripheral ulcerative keratitis, pigment deposits on the anterior lens capsule and posterior subcapsular cataract in the right eye

**Fig. 2:**
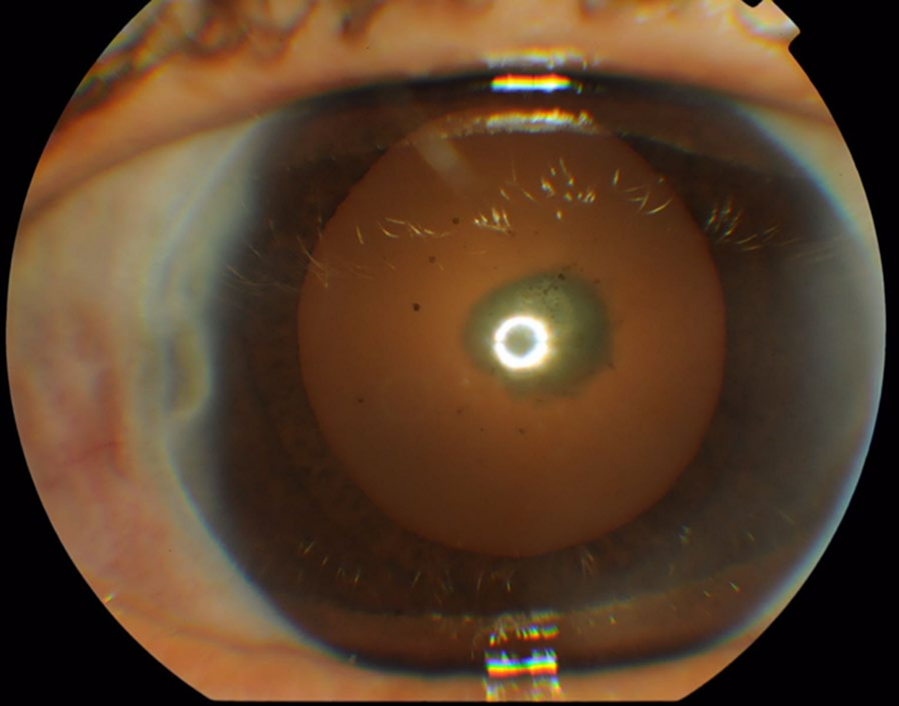
Nodular scleritis and peripheral stromal keratitis in the left eye

On examination, visual acuity was 5/10 right and 3/10 left unaided. Both eyes, seen in natural light, had a dark coloration of the sclera typical of the scleromalacia. Slit lamp biomicroscopy of the left eye revealed dilatation of the deep episcleral vascular plexus and scleral edema distinguished in a nodule adjacent to the temporal limbus, measuring around 3 × 3 mm in size, firm, immobile, and tender to palpation (Fig. 1). The peripheral cornea was seat of a crescent-shaped white-grayish opacity located 2 mm from the limbus with intact overlying epithelium and thinned and infiltrated underlying stroma; which strongly suggested the presence of cicatrised peripheral ulcerative keratitis. There were also some foci of stomal infiltrates. The examination of the left eye also found a circumciliary congestion, + 2 cells and + 1 flare anterior chamber activity, an eccentric and irregular pupil, and a pseudophakic posterior chamber with opacification and phimosis of the anterior capsule. The examination of the right eye found similar corneal lesions, an optically empty anterior chamber, pigment deposits on the anterior lens capsule, and posterior subcapsular cataract (Fig. 2). Intraocular pressure (Goldman tonometer) was 14 mmHg right and 10 mmHg left eye respectively. Fundoscopy was normal in both eyes, as well as fluorescein angiography and ultrasound B-scan. On the basis of these features, the patient was diagnosed with active nodular non necrotizing scleritis-associated with anterior uveitis in the right eye and bilateral sequelae of previous episodes of sclerokeratitis and sclerouveitis.

The patient was hospitalized for etiologic inquiry. Systemic examination including respiratory system was reported normal. Laboratory investigation found an elevated erythrocyte sedimentation rate of 40 mm/h. The tuberculin skin test (TST) results showed a 20 mm induration and the QuantiFERON-TB Gold test was positive with value over 3 IU/ml. X-ray and CT scan of the chest revealed no specific pulmonary fibrotic lesions. Other investigations including routine haemogram, C-reactive protein, serum electrolytes, renal and hepatic functions, urine analysis, serum rheumatoid factor, antineutrophilic cytoplasmic antibodies (p and c), antinuclear antibodies, angiotensin converting enzyme, syphilitic serology (TPHA-VDRL), serologies for viral hepatitis (B and C), ELISA for human immunodeficiency virus (1–2), sacroiliac joint radiographs and X-ray of sinus were normal. Biopsy of the sclera couldn’t be attempted due to the high risk of eye damage. Based on the foregoing clinical information and investigation results, the patient was diagnosed with presumptive latent tuberculosis-related scleritis. The inflammatory episode was well controlled a week after use of a selective cox-2 inhibitor and a topical corticosteroid. In addition, the patient received, under the supervision of the pneumologist, the first-line four-drug anti-tubercular therapy including isoniazid 5 mg/kg/day, rifampicin 10 mg/kg/day, ethambutol 15 mg/kg/day, and pyrazinamide 25 mg/kg/day initially for 2 months. Thereafter, isoniazid and rifampicin were used for an additional 7 months. Pyridoxine supplementation was given until cessation of therapy. No recurrences were observed over 12 months of follow-up since completing therapy.

## Discussion

Scleritis is a severe inflammatory condition characterized by edema and cell infiltration of the sclera [4]. According to the classification of Watson and Hayreh, scleritis is divided into anterior and posterior types based upon the anatomic distribution of disease [5]. Anterior scleritis, the most common type [1], is further subdivided into diffuse, nodular, necrotizing with inflammation, and necrotizing without inflammation (scleromalacia perforans) [5]. These forms of anterior scleritis correspond roughly to different degrees of severity [2].

In eyes with scleritis, the inflammatory process may extend to adjacent structures, causing several complications that may lead to loss of vision [4]. Anterior scleritis can be associated with anterior uveitis, cataracts, glaucoma and peripheral keratopathy [1, 2, 4, 6]. Clinical types of peripheral keratopathy associated with scleritis include peripheral corneal thinning (intact epithelium and no inflammatory cells in the stroma), stromal keratitis (intact epithelium but with inflammatory cells in the stroma and without stromal ulceration, and peripheral ulcerative keratitis (epithelial defect, inflammatory cells in the stroma, and stromal ulceration) [6]. Specific complications of posterior scleritis can include exudative retinal detachment, optic disk edema, cystoid macular edema, and choroidal folds [1, 2]. Other common complications include scleral thinning and globe rupture with minor trauma [2].

Scleritis may be idiopathic or associated with local or systemic disease. Autoimmune conditions are found in approximately 40 % of patients and infections in approximately 7 % [2]. A large number of vasculitic and connective tissue diseases are associated with scleritis but the most common are rheumatoid arthritis and Wegener granulomatosis [1, 2]. Other less commonly associated systemic diseases include systemic lupus erythematosus, relapsing polychondritis, polyarteritis nodosa, inflammatory bowel disease, sarcoidosis, juvenile rheumatoid arthritis, ankylosing spondylitis, cryoglobulinemia, Cogan’s syndrome, systemic vasculitis, temporal and Takayasu arteritis [1, 2]. The most commonly associated infection is herpes zoster.

Tuberculosis (TB) is one possible infectious cause of scleritis and among other ocular inflammatory manifestations [7–12]. Ocular Mycobacterium tuberculosis (MTB) infection is most often a result of hematogenous dissemination from a distant site (such as lungs) [3, 9, 12]. Infection may also occur by direct extension from surrounding tissue or direct inoculation [9, 12]. Definitive diagnosis of ocular TB is based on demonstration of MTB in ocular samples, from polymerase chain reaction (PCR) detection, growth in cultures or detection of acid-fast bacilli on smears [13, 14]. Such tests are not sensitive [3, 9] and even the caseating granulomatous inflammatory lesion as histopathological evidence of ocular TB is rarely available [11, 14]. Ocular TB infection is difficult to diagnose due to the invasiveness of obtaining tissue samples, and limitations of available diagnostic tests [9]. Additionally, immune-mediated ocular TB can occur due to hypersensitivity to MTB antigens from a distant focus despite the absence of bacterium in the eye [3, 8, 9, 11, 12]. Unfortunately, there is no pathognomonic clinical manifestation for ocular TB [9]. Considering this features, the diagnosis of ocular TB remain largely presumptive, supported by the combination of corroborative evidences such as suggestive history and clinical signs, detection of MTB in non-ocular samples or positive indirect tests for TB infection, radiologic findings of either active or latent TB infection, exclusion of other etiologies and response to anti-tubercular treatment without relapse [10, 12–14].

TB-related ocular inflammation mainly accompanies latent TB (LTB) [14], wherein the patient is infected with MTB but does not have active TB disease [9, 11, 14]. It is estimated that LTB affect one-third of the world’s population and about 10 % of patients with LTB will go on to develop active TB at a later stage of their life [9, 14]. Progression of LTB to active TB is more common among people with compromised immune systems and those with certain medical conditions [9] as diabetes present in our patient. In Morocco, country where TB is endemic with over 27,000 new cases detected annually, LTB as a possible etiology of scleritis and other ocular inflammations is often ignored. In the absence of others presumed etiologies, such manifestations continue to be labeled idiopathic and are often treated with topical corticosteroids and/or non-steroidal anti-inflammatory drugs. Systemic steroids and immunosuppressive drugs can also be used to manage the disease, and this may have catastrophic consequences in patients with undiagnosed LTB infection [3, 9, 11].

The methods currently available to detect LTB are the tuberculin skin test (TST) and the interferon gamma assays release assays (IGRAs) [9, 11]. The TST is the oldest and most widespread test used for tuberculosis diagnosis [9]. Its drawbacks are operator-dependent placement and reading. Sometimes, a history of BCG vaccination can yield false-positive results due to a cross-reaction with *Bacillus* Calmette-Guérin (BCG) that contains a strain of the closely related bacteria, Mycobacterium bovis, but in our case, given the interval between the vaccination and the TST, it seems unlikely [9, 15]. IGRAs, such as QuantiFERON-TB Gold, are in vitro tests that measures IFN-γ released when whole blood is stimulated with 2 synthetic peptides, the early secreted antigenic target 6-kDa protein (ESAT-6) and the 10-kDa culture filtrate protein (CFP-10), both found in MTB but not in the BCG vaccine or in the vast majority of atypical mycobacterium [15]. However, the QuantiFERON-TB gold is expensive and most patients in developing countries cannot afford it. Thus, QuantiFERON-TB Gold is more specific for LTB infection than the tuberculin skin test in a population recently vaccinated by BCG, but the TST gives a better cost-effectiveness pattern [14–16]. In the present case, the TST result was positive with induration large enough to raise our suspicion of TB. The QFT testing strengthened the diagnosis.

Ocular TB is treated with medical anti-tuberculosis treatment (ATT) similar to that for pulmonary TB [13]. The CDC recommends the use of all four drugs (isoniazid, rifampicin, pyrazinamide, and ethambutol) for an initial 2-month period followed by a choice of different options over the next 4–7 months. A low dose steroid given concomitantly with anti-tubercular therapy drugs for 4–6 weeks has been shown to have a protective effect against tissue damage from delayed hypersensitivity [7, 10, 13, 14]. In patients with LTB-related ocular inflammation, the administration of ATT would lead to reduce ocular inflammation and its futures recurrences as the inciting MTB load is reduced [13]. All of these decisions should be made in collaboration with an infectious disease specialist.

## Conclusions

Tuberculosis, including latent form, is a possible infectious cause of scleritis and other ocular inflammatory manifestations. Delayed diagnosis can lead to vision loss and systemic complications of the infection. Tuberculosis-related ocular inflammation is a challenging clinical entity that may be difficult to diagnose and manage. However, a favorable response to ATT without relapse is taken as evidence of the disease.

## Abbreviations

NSAID: non-steroidal anti-inflammatory
TB: tuberculosis
MTB: mycobacterium tuberculosis
TST: tuberculin skin test
IGRA: interferon gamma assays release assays
LTB: latent tuberculosis
ESAT-6: early secreted antigenic target
CFP: culture filtrate protein
ATT: anti-tuberculous treatment
